# Healthcare utilisation in the United Arab Emirates for children with attention-deficit hyperactivity disorder and comorbidities

**DOI:** 10.1192/bji.2023.14

**Published:** 2023-08

**Authors:** Ahmad M. Almai, Jay A. Salpekar

**Affiliations:** 1 Consultant child and adolescent psychiatrist; Director of Residency Training, Department of Psychiatry, Johns Hopkins Aramco Health Center, Dhahran, Kingdom of Saudi Arabia. Email ahmed.almai@jhah.com; 2 Director of the Neuropsychiatry Center, Kennedy Krieger Institute, Baltimore, Maryland, USA; Associate Professor of Psychiatry and Neurology, Department of Psychiatry, Johns Hopkins University School of Medicine, Baltimore, Maryland, USA

**Keywords:** ADHD, comorbidity, children, UAE, service delivery

## Abstract

The prevalence of attention-deficit hyperactivity disorder is consistent worldwide. Psychiatric comorbidities are common, although less is known about how those comorbidities affect utilisation of healthcare services. Access to paediatric mental healthcare is a challenge in many regions. However, access to care in the United Arab Emirates (UAE) is supported by a well-established healthcare infrastructure with widely available primary care physicians. A review of diagnosis codes suggests that a clear correlation exists between the number of comorbidities and increased utilisation of available mental health services. Infrastructure in the UAE may represent a successful model for paediatric mental healthcare.

Attention-deficit hyperactivity disorder (ADHD) has long been recognised as the most frequently diagnosed behavioural disorder of childhood and is characterised by a persistent pattern of inattention and/or hyperactivity–impulsivity that interferes with functioning or development. Comorbidity is common: a study of children with ADHD in the USA found that 67% had at least one other parent-reported mental health or neurodevelopmental disorder, with 18% having three or more comorbid conditions.^1^ A Swedish study found that 87% of children with ADHD met the criteria for having another disorder and 67% met the criteria for two or more.^2^ Oppositional defiant disorder may be particularly common, with a prevalence of 54–84% worldwide.^3^

Children with ADHD, with or without other psychiatric comorbidities, use more general medical services than do other groups of children, including out-patient, emergency or urgent care visits. ADHD with psychiatric comorbidities also leads to higher use of specialty mental health services and greater use of psychotropic medications.^4^ Literature primarily published in the USA shows that children with ADHD have higher medical costs, being 9.02 and 8.75 times more likely than other children to have out-patient mental health visits and pharmacy refills (prescriptions) respectively.^5^

A strong genetic component for ADHD has been demonstrated by family, twin and adoption studies.^6^ This is important in the United Arab Emirates (UAE) and specifically in Abu Dhabi because of the high prevalence of hereditary disorders, possibly attributable to high rates of consanguinity.^7^ Intellectual and developmental disabilities, including autism spectrum disorders, are also suspected to have some genetic components overlapping with ADHD.^8^ Consanguinity makes genetic diatheses potentially over-represented in the UAE and in the case of ADHD, comorbidity would be expected to be more complex and to yield increased demand for paediatric mental health services.

## The Emirate of Abu Dhabi

The Emirate of Abu Dhabi is one of seven emirates that constitute the UAE. Abu Dhabi has the second largest population of the seven. In June 2011, it was estimated to be 2 120 700 people, of whom 439 100 (less than 21%) were Emirati citizens.^9^ Abu Dhabi Health Services Company (SEHA) is the government department charged with providing healthcare services to the UAE nationals and other residents in the Emirate of Abu Dhabi. SEHA provides services in 12 hospitals and 65 clinics, all connected through one electronic health records system. Children are typically seen at primary care clinics before being referred to psychiatry and other specialties for further care. The organisation of healthcare delivery in the UAE is personalised and accessible to multiple providers, and is rapidly becoming viewed as high quality, even on a par with many Western medical systems.

Information gathered from all SEHA facilities with psychiatry, paediatrics, primary care, neurology and other medical services for children affirms the high-quality infrastructure. For example, queries can be readily made of the SEHA Electronic Health Record database. Data on number and type of visits as well as patient age, gender and nationality can be assessed. Coding data are available for many associated psychiatric conditions, including conduct disorder, autism spectrum disorders, language/speech disorders, intellectual disabilities, bipolar disorder, anxiety disorder, enuresis and encopresis, tics and movement disorders, depressive disorders and other psychiatric illnesses. To assess service delivery, we made a cursory query for all children aged 0–17 years with an ICD-9 diagnostic code of ADHD (codes from 314.00 to 314.9). This report made use of statistical data fully anonymised in the context of institutional quality assurance activities. Institutional Review Board and ethics committee approval was obtained for the database review/quality assurance analysis (approval #REC-27.02.2013 (RS-240)).

## Patterns of healthcare utilisation

During a representative 4-year period from approximately 2008 to 2012, data from electronic medical records show that 1301 children had a diagnosis of ADHD and received 16 371 total clinical visits. Although more boys were present in the sample (77.2%), that is consistent with the typical gender distribution for clinical presentation of ADHD in childhood. The age range was 35.8% (*n* = 466) aged 5 or under, 51.1% (*n* = 665) aged 6–12 and 13.1% (*n* = 170) aged 13 years or over, also skewed towards primary school age, similarly consistent with typical presentations of ADHD. There was no difference in age distribution across genders (*P* = 0.75): the mean age of boys was 7.4 years (s.d. = 4.19) years and that of girls was 7.5 years (s.d. = 3.93). Approximately 53% of the sample were either UAE or Gulf country nationals, and the remainder had other national origins. The mean number of clinical encounters per child was 12.6, with a clear relationship between number of comorbidities and a higher number of clinical visits.

## Comorbid mental disorders

**Fig. 1:**
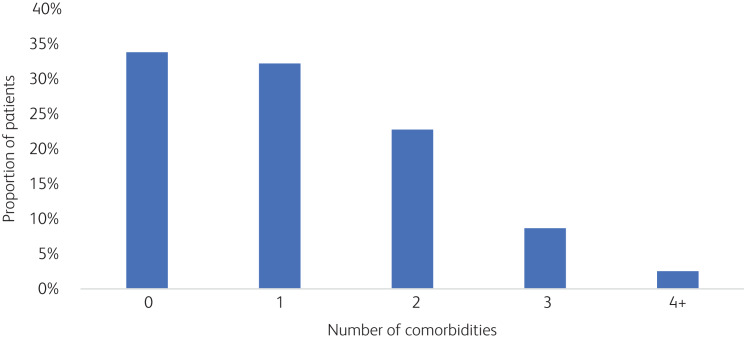
Patients with attention-deficit hyperactivity disorder and comorbidities.

**Fig. 2:**
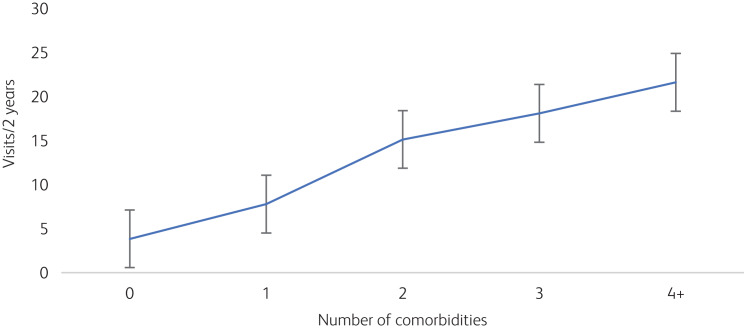
Impact of comorbidities on number of healthcare visits over a 2-year period. The error bars show 95% confidence intervals.

Among this cohort of children with ADHD, several behavioural and mental conditions had been recorded (Fig. 1). Referral patterns appeared to have allowed identification of comorbidities at a level mostly consistent with what is seen worldwide. Roughly one-third of the children had no additional behavioural comorbidity, one-third had one comorbidity and one-third had two or more comorbidities. Language/speech disorders were the most frequently occurring comorbid conditions, which were followed by intellectual disability and autism spectrum disorder. Children with more comorbidities clearly tended to have more healthcare visits (Fig. 2). This was true for the whole cohort as well as for Emirati nationals alone.

## Discussion

ADHD was clearly common in the UAE paediatric population, and consistent with worldwide prevalence, it affected males more frequently than females, with a ratio of over 3:1. In the population of children seen at SEHA facilities over the observed period, the majority of those diagnosed with ADHD were in the age group of 6–12 years. These results are validating, given their consistency with observed prevalence of comorbid conditions in children with ADHD in other countries.

This perspective is important for several reasons. As mentioned above, ADHD has a strong genetic component, with hereditary attribution of up to 90%.^6^ Hereditary disorders are important in the UAE, which has a high rate of consanguinity. Although this region is understudied in terms of mental healthcare utilisation, the level of infrastructure has improved in quality, given the deliberate investment in service delivery. The quality of the electronic medical records shows that the prevalence of ADHD and comorbid conditions are readily identifiable even in this region of the world. Furthermore, the correlation between the numbers of comorbidities and utilisation of health services is reassuring, given its consistency with other high-quality healthcare services worldwide. This observation suggests that an organised system with a single accessible medical record for each patient and primary care-driven referrals may be effective in providing mental healthcare in paediatrics even in a region of the world not traditionally accustomed to delivery of those services.

Despite this encouraging perspective, important caveats should be noted. The usage of medical record coding and physician diagnosis could lead to under- or overdiagnosis of comorbidities, although this limitation is not unique to the UAE. A strength of this system is the access to the complete set of data from Abu Dhabi's largest healthcare system. As an institution, SEHA's catchment includes approximately 85% of the population,^10^ thus it is likely that the information available allows analyses that accurately predict the national resources needed to manage ADHD and comorbidities. In that way, the Emirate of Abu Dhabi could prove to be a strong example of how mental healthcare services may ideally be developed and then adjusted based on demand.

## Conclusions

Ultimately, the clear and positive message is that regardless of culture or geographical location, a well-organised healthcare system appears to allow appropriate levels of healthcare utilisation even for complex comorbidities associated with paediatric mental illness. The correlation between the number of comorbidities and increased utilisation of health services shows that the system works even for those with language/speech disorders, intellectual disabilities and autism spectrum disorders. These observations can be used to guide investment in clinical resources for the assessment and management of ADHD and complex mental health conditions in other populations. Outcome studies in this or in similar systems could further elucidate the effectiveness of different types of infrastructure that may comprehensively guide treatment for complex paediatric mental health conditions.

## Data availability

Restrictions apply to the availability of these data, which were used with the permission of Abu Dhabi Healthcare Services, as requested by the corresponding author, A.A.

## Author contributions

A.A. designed the study and performed the initial data analysis. A.A. and J.A.S. substantially interpreted data, wrote and revised the manuscript and approved the final drafts of the manuscript.
